# N‑Terminal Protein Complexation and Assembly with a Triangular Sulfated Macrocycle

**DOI:** 10.1021/acs.cgd.5c00290

**Published:** 2025-04-10

**Authors:** Marvin C. Ifeagwu, Lijuan Guo, Niamh M. Mockler, Ming Dong, Chunju Li, Peter B. Crowley

**Affiliations:** † School of Biological and Chemical Sciences, University of Galway, Galway H91 TK33, Ireland; ‡ Tianjin Key Laboratory of Structure and Performance for Functional Molecules, College of Chemistry, 12523 Tianjin Normal University, Tianjin 300387, P. R. China

## Abstract

We report two cocrystal structures of a mutant *Ralstonia solanacearum* lectin (RSL) in complex with the recently described sulfated terphen[3]­arene (**STP3**). This triangular macrocycle bearing 12 sulfates exhibits interesting protein-binding modes including methionine encapsulation and insertion between surface-exposed loops. These two binding modes facilitate the overall crystal packing, which may benefit from the macrocycle rigidity. In addition to a promising tool for protein assembly and crystallization, the data have implications for lectin–heparan sulfate interactions.

## Graphical abstract

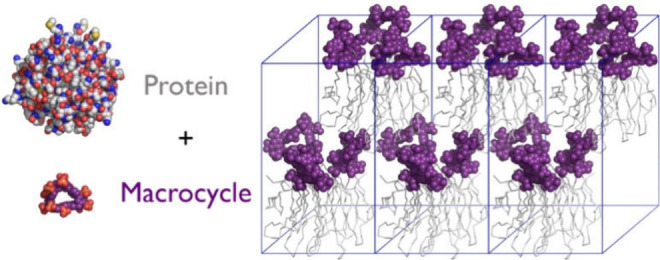

Research at the interface of protein science and supramolecular chemistry is yielding interesting discoveries in molecular recognition, self-assembly and encapsulation.^1–5^ Supramolecular strategies of protein assembly may prove useful for fabricating biomaterials.^4,5^ Here, we report cocrystal structures of a large anionic macrocycle and a lectin, revealing novel modes of N-terminal recognition and solid-state assembly.

Sulfated terphen­[3]­arene (**STP3**) is a large, rigid, triangular macrocycle comprising terphenyl sides, methylene vertices, and 12 sulfate substituents (Figure 1).^6,7^ Owing to the sulfates, **STP3** has high water solubility and a low propensity to self-associate (as evidenced by ^1^H NMR in D_2_O). Similar to anionic cyclodextrins^8^ and sulfated pillarenes,^9^ **STP3** has high affinity for hydrophobic cations such as the aminosteroid rocuronium (*K*_a_ = 10^8^ M^–1^).^7^ We were interested in testing **STP3**–protein interactions. Ongoing protein–macrocycle cocrystallization studies demonstrate the versatility of sulfonato-calix[8]­arene (**sclx**_**8**_) for assembly, including the formation of (porous) protein–**sclx**_**8**_ frameworks.^4^ *Ralstonia solanacearum* lectin (RSL) is a case in point with four polymorphs identified to date.^4,10,11^ RSL is a neutral trimer with a toroidal, β-propeller fold. Certain RSL–**sclx**_**8**_ cocrystals only grow at pH values below the isoelectric point of the protein (p*I* ∼ 6.5). Low pH enables protein–macrocycle Coulombic attraction, and favors **sclx**_**8**_ dimerization. Considering the broad similarities in size and net charge of **sclx**_**8**_ and **STP3**, the latter is a candidate for protein assembly. The differences in conformation and dynamics of these two macrocycles are of interest. **sclx**_**8**_ with 8 methylene bridges is flexible and can mold to a protein surface.^4,10^ In contrast, **STP3** with terphenyl sides is rigid, which may impose constraints on protein binding. We studied RSL–**STP3** interactions by cocrystallization and X-ray crystallography.

**Figure 1:**
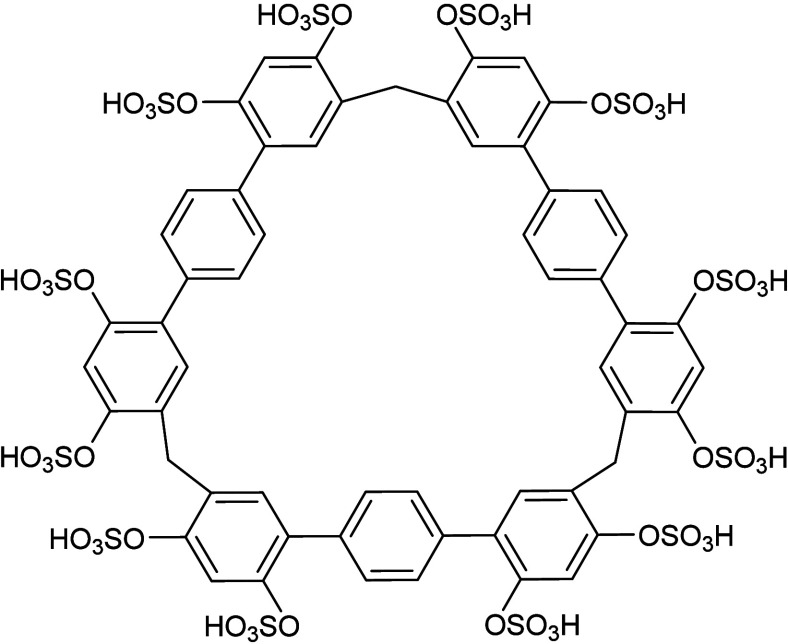
Molecular structure of **STP3** in the acid form (1880 Da).

The sodium salt of **STP3** was prepared as a ∼0.1 M aqueous solution at pH ∼7. Mixtures comprising 1 mM protein and 1 or 5 mM macrocycle were tested in a sparse matrix cocrystallization screen (JCSG++ HTS, Jena Bioscience) dispensed with an Oryx8 robot (Douglas Instruments). The screen did not yield any leads for RSL and **STP3**. We then turned to the mutant MK-RSL, which contains a Met-Lys motif at the N-terminus of RSL. This sterically accessible and dicationic site functions as a macrocycle binding tag.^1,12^ A cocrystallization screen of MK-RSL and **STP3** yielded rod-shaped crystals (Figure 2a) in conditions comprising 5 mM macrocycle, 20–25% PEG 3350, with or without 0.1 M Bis-Tris pH ∼6, and 0.2 M salt (ammonium formate, ammonium acetate or sodium chloride). We also obtained hexagonal cocrystals (Figure 2b) in the simple precipitant/buffer system, ∼1 M sodium citrate at pH 4. Previously, a citrate pH 5–6 condition yielded one of the RSL–**sclx**_**8**_ polymorphs.^10^

**Figure 2:**
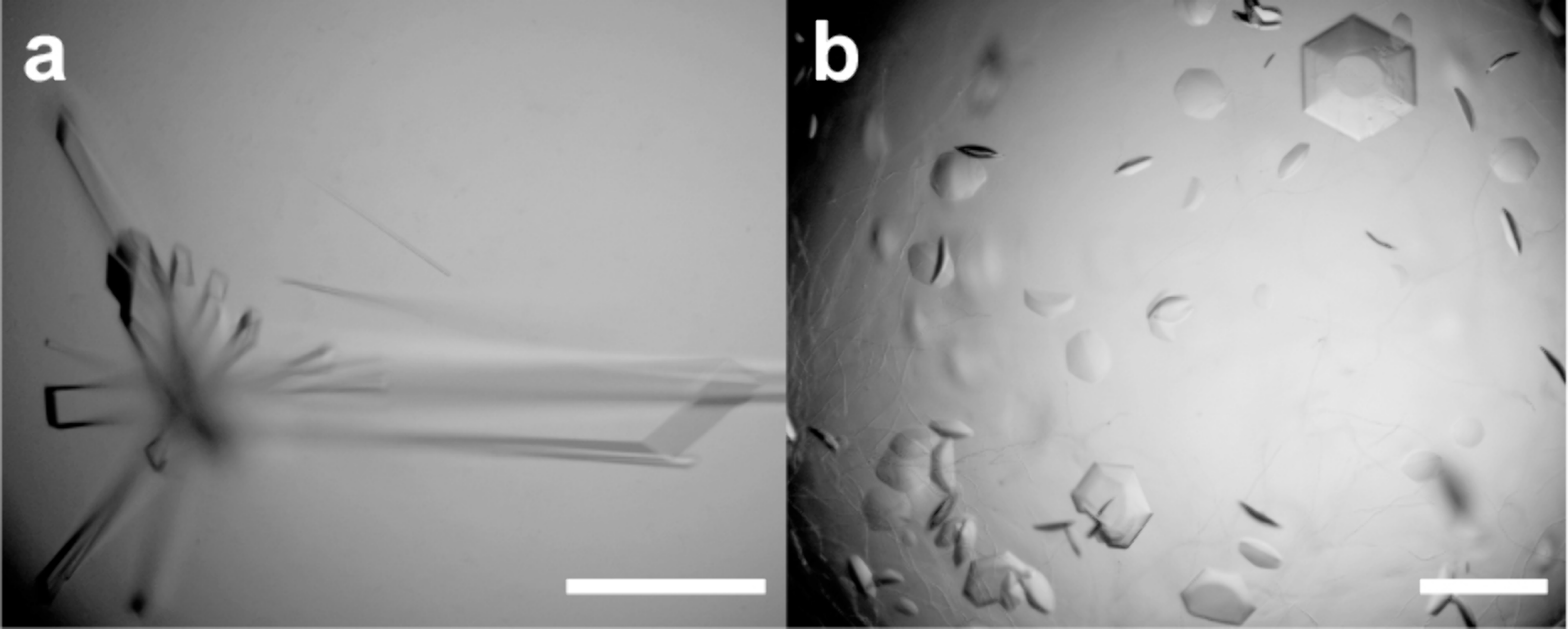
Cocrystals of **STP3** and MK-RSL grown in (a) 25% PEG 3350, 0.2 M NaCl, 0.1 M Bis-Tris pH 5.5, and (b) 1.2 M sodium citrate at pH 4. Scale bars are 250 μm.

We tested the two types of MK-RSL–**STP3** cocrystals at SOLEIL synchrotron. Table 1 lists the X-ray data collection, processing and refinement statistics. The structures were solved, refined and analyzed using standard methods^4,10^ and deposited in the Protein Data Bank. The MK-RSL–**STP3** cocrystals grown in PEG (Figure 2a) diffracted to a maximum resolution of 1.6 Å. The structure in space group *C*121 has an asymmetric unit comprising two MK-RSL trimers (6 protein chains). Only one molecule of **STP3** is present and is located close to a *C*_2_ symmetry axis. The macrocycle and the interfacing protein surface are partially disordered. Clear electron density is evident for only one vertex of the macrocycle that complexes Lys1 in one protein chain. The adjacent N-terminal methionine (Met0) is disordered. Owing to the degree of disorder in this structure, we do not consider it further for characterizing **STP3**–protein interactions.

**Table 1:** X-ray Data Collection, Processing and Refinement Statistics for the MK-RSL–**STP3** Cocrystals

**Crystallization**		
**Precipitant**	25% PEG 3350	1.2 M Na citrate pH 4.0
**Buffer (0.1 M)**	Bis-Tris pH 5.5	-
**Additive (0.2 M)**	NaCl	-

**Data Collection**		
**Light Source**	SOLEIL, PROXIMA-2A	
**Wavelength (Å)**	0.97856	
**Space group**	*C*121	*P*6_3_
**Cell constants** **α, β, λ (°)**	90, 108.236, 90	90, 90, 120
**Cell constants** ***a***, ***b***, ***c*** **(Å)**	167.523, 45.994, 70.159	43.526, 43.526, 85.907
**Resolution (Å)**	44.664–1.643 (1.671–1.643)	37.695–1.696 (1.725–1.696)
**# reflections**	430473 (15406)	193440 (7271)
**# unique reflections**	61998 (2828)	10257 (505)
**Multiplicity**	6.9 (5.4)	18.9 (14.4)
**I/σ (I)**	11.0 (1.8)	19.9 (2.9)
**Completeness (%)**	99.6 (92.0)	99.9 (98.2)
***R***_**meas**_ **(%)**	12.2 (86.9)	10.2 (81.0)
***R***_**pim**_ **(%)**	4.6 (35.7)	2.3 (20.5)
**CC**_**1/2**_	0.998 (0.777)	0.999 (0.971)
**Solvent content (%)** ^a^	41	38

**Refinement**		
***R***_**work**_	0.156	0.156
***R***_**free**_	0.194	0.193
**rmsd bonds (Å)**	0.009	0.019
**rmsd angles (°)**	0.952	1.324

**# Molecules in Asymmetric Unit**		
**Protein chains**	6	1
**STP3**	1	1
**water**	629	85
**Ave. B-factor (Å** ^**2**^ **)**	17.41	21.85
**Clashscore**	1.64	1.94

**Ramachandran Analysis, % Residues**		
**in favored regions**	97.75	96.63
**in allowed regions**	2.25	3.37

**PDB code**	9I1O	9I1N

a Matthew’s calculation, accounting for the sum of protein and macrocycle masses.

The MK-RSL–**STP3** cocrystals grown in sodium citrate at pH 4 (Figure 2b) diffracted to 1.7 Å and the structure was solved in space group *P*6_3_ with one protein chain. One clearly defined **STP3** is also present in the structure (Figure 3a). The ∼2 kDa and highly anionic macrocycle bridges two protein molecules by a combination of binding modes. One protein chain interacts with the macrocycle via N-terminal methionine encapsulation. *Circa* 180 Å^2^ of Met0 is buried in the complex with **STP3**. The thioether side chain sits in the macrocycle cavity, forming CH−π interactions with three phenyl rings (Figure 3a),^12^ reminiscent of methionine–aromatic interactions in protein tertiary structures.^13^ The thioether sulfur makes van der Waals contacts with one phenyl ring. Surprisingly, the N-terminal ammonium group points toward the solvent rather than forming a salt bridge with one of the **STP3** sulfates. Similarly, the ammonium-containing side chain of Lys1 is partly disordered (solvated) rather than forming a salt bridge with the macrocycle. The Lys1 amide forms a weak hydrogen bond with one of the sulfates (Lys1–N^α^···^–^OSO_3_R = 3.0 Å, Figure 3a). Several additional polar residues, including Ser2, Asn23, Asp46 and Thr69, contribute to this interface with **STP3**.

**Figure 3:**
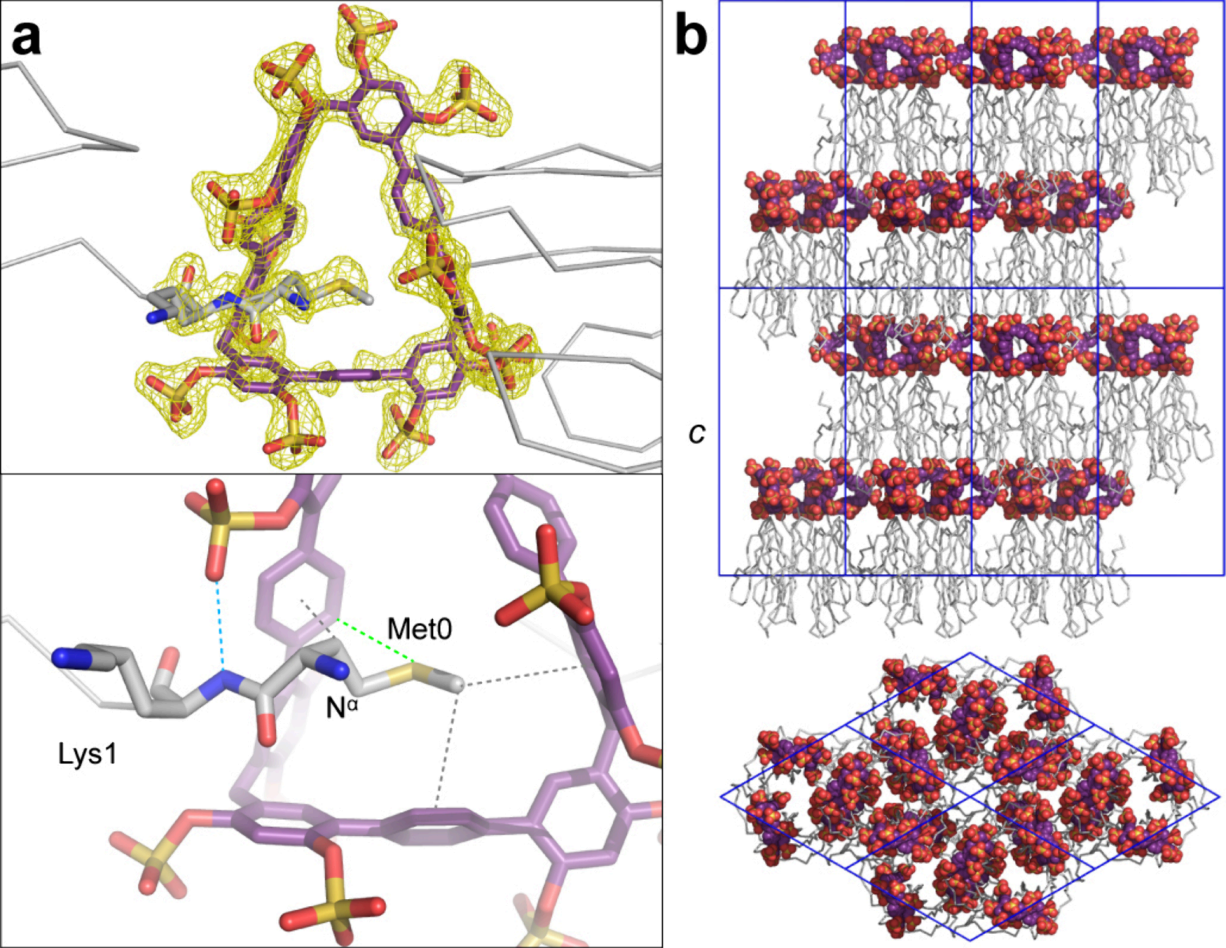
MK-RSL and **STP3** cocrystal structure. **(a) STP3** acts like *molecular glue* by encapsulating the N-terminus of one protein and slotting between two loops of another protein. The yellow mesh corresponds to the refined 2*F*_o_ – *F*_c_ electron density maps (contoured at 1.0σ) for **STP3**, Met0 and Lys1. For clarity, only two MK-RSL monomers are shown. In the lower panel, dashed lines indicate putative noncovalent interactions: CH−π (gray), S−π (green) and hydrogen bond (blue). N^α^ denotes the N-terminal ammonium. (b) Crystal packing in the *P*6_3_ structure, with the protein represented as the C^α^ ribbon and **STP3** as spacefill. Upper and lower images related by 90° rotation. Unit cell axes are blue. The *c* axis is ∼9 nm.

The other protein bound to **STP3** interacts in an altogether different way. Here, two loops of the β-propeller fold engulf one side of **STP3** (Figures 3a and 4). This binding mode is unprecedented in protein–synthetic macrocycle complexes. *Circa* 470 Å^2^ of the macrocycle is buried in the interface, and all four sulfates on this side of **STP3** form noncovalent bonds with the protein. Thr12 and Ser57 on adjacent engulfing loops appear to dominate the interaction, each contributing 70 Å^2^ to the interface. The carboxylate of Asp32, previously identified as a pH trigger in calixarene-binding,^4^ is within polar bond distance of two sulfates. Hydrogen bonds from sulfates to the protein backbone, involve the amide NH groups of Gly11, Thr12 and Val13. Also of note are two Tryptophan side chains. The indole of Trp10 forms an edge-to-face π–π interaction with **STP3**, while the indole of Trp76 forms a weak hydrogen bond with a sulfate. This protein–**STP3** interface also has hydrophobic contributions from the side chains of Val13, Leu54 and Ile59, including van der Waals contacts with sulfates.

**Figure 4:**
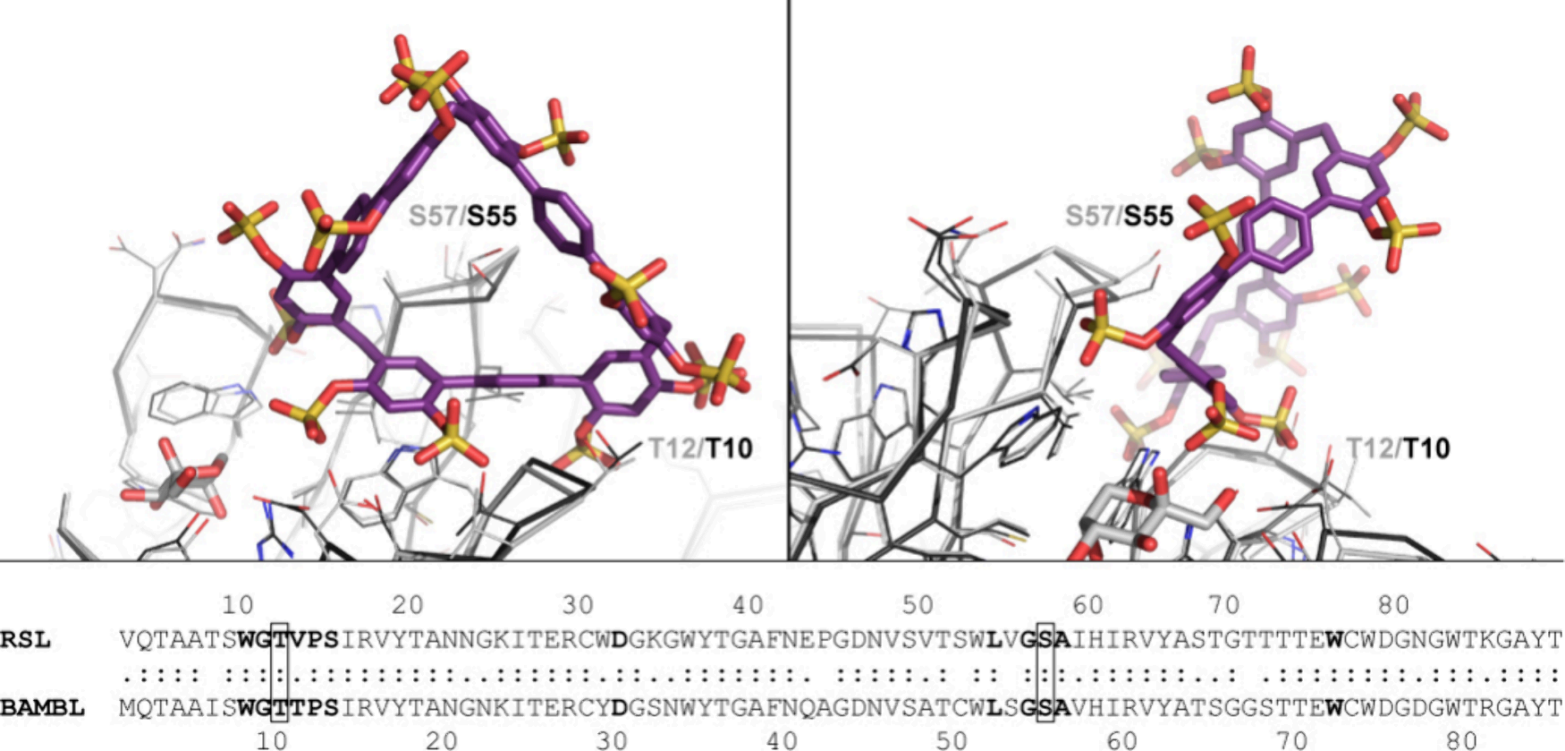
Superposition of the MK-RSL–**STP3** cocrystal structure with BAMBL (PDB 6ZFC) reveals a similar binding site between loops 2 and 5 in both proteins. Labels indicate loop residues T12/T10 and S57/S55. Proteins (MK-RSL in gray, and BAMBL in black) shown as C^α^ ribbons with side chains as lines. One β-D-fructose bound to MK-RSL is shown as sticks. Left and right panels related by a 90° rotation. The aligned sequences of RSL and BAMBL showing conserved residues (:) and the **STP3** binding site in bold.

The **STP3** binding site between two loops on the RSL β-propeller fold is suggestive of a binding pocket for heparan sulfate. Like **STP3**, heparan contains relatively rigid, sulfated segments (e.g., PDB 8v58).^14^ RSL is homologous with the fucose-binding lectin BAMBL from *Burkholderia ambifaria*, a soil bacterium and an opportunistic human pathogen.^15^ Sequence alignment (Figure 4) shows 77% identity in an 85-residue overlap of RSL and BAMBL. The cocrystal structure of MK-RSL and **STP3** when superposed with BAMBL reveals conservation of the **STP3** binding site in BAMBL. These observations raise the question of BAMBL–heparan interactions by a similar binding mode.

Interesting features occur in the MK-RSL–**STP3** crystal packing. Before discussing the overall packing, we consider the oligomeric structure and symmetry of the protein. RSL is a toroidal trimer with a wide end and a narrow end. The three N-termini are collocated at the narrow end. Each N-terminus is encapsulated by one molecule of **STP3**, such that each protein trimer bears three macrocycles. The crystal packing in space group *P*6_3_ involves honeycomb layers of MK-RSL interspersed with discrete layers of macrocycle (Figure 3b). One layer of protein interacts with the macrocycle layer through N-terminal encapsulation. The other layer of protein interacts via loops that engulf one side of the macrocycle. The interlayer crystal packing is mediated exclusively by the macrocycle, which apparently functions as both a *molecular glue* and a spacer. While the structure has an overall solvent content of 38%, there are distinct protein layers and macrocycle layers with varying porosity.^16^ The rigidity of **STP3**, with terphenyl sides, may be instrumental to the crystal packing (Figure 3b). Previously, we reported a cocrystal structure of MK-RSL and **sclx**_**8**_ in which the N-terminal Met-Lys motif is disordered (PDB 8C9Y).^10^ Apparently, the flexible calix[8]­arene cannot trap the N-terminus. In contrast, the rigid bowl of calix[4]­arene encapsulates the N-terminal methionine as observed in the MK-RSL–**sclx**_**4**_ cocrystal structure (PDB 9GR3).^12^ Now, with the rigid **STP3** we again see encapsulation of the N-terminal methionine (Figure 3a).

In conclusion, two cocrystal structures of a protein and the heavily sulfated terphen[3]­arene reveal new features of biomolecular recognition and assembly. The cocrystals grown in PEG (low salt) include only one **STP3** per 6 protein monomers and the protein–macrocycle interfaces are partly disordered. Crystals grown in high salt and low pH, yield a well-defined complex in which each of the N-termini of MK-RSL are encapsulated by a macrocycle. The low pH, rendering the protein cationic, the involvement of the dicationic N-terminus (Met0Lys1) and the likely protonation of Asp32,^4^ all point to the importance of charge–charge interactions. However, the high ionic strength arising from ∼1 M citrate dampens the Coulombic attraction between the cationic protein and anionic macrocycle. The consequent salting out effects (Hofmeister) apparently favor cocrystal formation in a segregated fashion with anionic macrocycle layers interspersing cationic protein layers. On one side of the macrocycle layer, proteins are engaged by polar loops as well as contributions from tryptophan and aliphatic side chains. On the other side of the macrocycle layer, protein binding focuses on the N-terminal tag. Apparently, the rigidity of the macrocycle is advantageous to trapping the N-terminus.^12^ The ∼2 kDa rigid macrocycle almost entirely encapsulates the N-terminal methionine. Such specificity arising through interaction with a single residue is rare in protein–ligand binding.^1^ Future studies will determine the broader applicability of the **STP3**–N-terminal methionine interaction for protein assembly.

## Notes

PDB entries 9I1N and 9I1O contain supplementary crystallographic data, available free of charge from the Protein Data Bank.
